# Health-related quality of life in persons post-COVID-19 infection in comparison to normative controls and chronic pain patients

**DOI:** 10.3389/fpubh.2022.991572

**Published:** 2022-10-20

**Authors:** Maarten Moens, Rui V. Duarte, Ann De Smedt, Koen Putman, Jonas Callens, Maxime Billot, Manuel Roulaud, Philippe Rigoard, Lisa Goudman

**Affiliations:** ^1^STIMULUS Research Group (reSearch and TeachIng neuroModULation Uz bruSsel), Vrije Universiteit Brussel, Brussels, Belgium; ^2^Department of Neurosurgery, Universitair Ziekenhuis Brussel, Brussels, Belgium; ^3^Center for Neurosciences (C4N), Vrije Universiteit Brussel, Brussels, Belgium; ^4^Pain in Motion (PAIN) Research Group, Department of Physiotherapy, Human Physiology and Anatomy, Faculty of Physical Education and Physiotherapy, Vrije Universiteit Brussel, Brussels, Belgium; ^5^Department of Radiology, Universitair Ziekenhuis Brussel, Brussels, Belgium; ^6^Liverpool Reviews and Implementation Group, University of Liverpool, Liverpool, United Kingdom; ^7^Department of Physical Medicine and Rehabilitation, Universitair Ziekenhuis Brussel, Brussels, Belgium; ^8^Department of Public Health (GEWE), Faculty of Medicine and Pharmacy, Interuniversity Centre for Health Economics Research (I-CHER), Vrije Universiteit Brussel, Brussels, Belgium; ^9^PRISMATICS Lab (Predictive Research in Spine/Neuromodulation Management and Thoracic Innovation/Cardiac Surgery), Poitiers University Hospital, Poitiers, France; ^10^Department of Spine Surgery & Neuromodulation, Poitiers University Hospital, Poitiers, France; ^11^Pprime Institute UPR 3346, CNRS, ISAE-ENSMA, University of Poitiers, Chasseneuil-du-Poitou, France; ^12^Research Foundation—Flanders (FWO), Brussels, Belgium

**Keywords:** health-related quality of life, EQ5D-3L, chronic pain, COVID-19, burden of disease

## Abstract

The Coronavirus disease 2019 (COVID-19) pandemic exerted a tremendous pressure on the healthcare system, people's social life, mental health and financial status with profound implications for the general population. The exact impact of the pandemic on the overall physical, mental and social wellbeing of COVID-19 infection survivors on the long term has not yet been explored in a thorough way. Based on the reporting of persistent pain, fatigue and dyspnea symptoms by these survivors, it is our hypothesis that their quality of life will be extremely impacted, as is observed in patients with chronic pain. Therefore, the first aim of this study was to perform an in-depth evaluation of the quality of life of post-COVID-19 infected persons. The second aim was to compare the quality of life of these persons with a normative population and with patients with chronic pain. Health-related quality of life, as a measure for a person's overall physical, mental, and social wellbeing, was measured with the 3-level EQ5D in 547 post-COVID-19 infected persons. These data were compared to reference data from normal population records for Belgium and to data from patients with chronic pain after spinal surgery with two-way analyses of variance. In total, 89.58% of the post-COVID-19 infected persons reported pain/discomfort and 82.45% indicated limitations when performing usual activities, when evaluated 287 days (SD: 150) after the infection. Self-care was preserved in most post-COVID-19 persons, whereby only 13.16% indicated problems. The mean EQ5D-3L index score was 0.57 (SD: 0.23) and EQ5D VAS mean score was 56.6 (SD: 18.2). The mean index score for the normative population was significantly higher than for COVID-19 infected persons [mean difference of 0.31 (95% from 0.29 to 0.33), *p* < 0.01] while the mean score of chronic pain patients was significantly lower than the score of COVID-19 infected persons [mean difference of −0.31 (95% from −0.29 to −0.33), *p* < 0.01]. Compared to age-and sex adjusted reference data, health-related quality of life of persons with long COVID is severely impacted. In relation to patients with chronic pain after spinal surgery, the quality of life of post-COVID-19 infected persons seemed to be better.

**Clinical trial registration:**

https://www.clinicaltrials.gov/, identifier: NCT04912778.

## Introduction

Coronavirus disease 2019 (COVID-19), a highly infectious disease caused by the novel Severe Acute Respiratory Syndrome (SARS) Coronavirus-2, was first reported in China in 2019 (1) and rapidly expanded into a worldwide pandemic. Characteristic clinical manifestations of this disease include fever, cough, fatigue, dyspnoea, sore throat, and myalgia (2). Due to the limited availability of appropriate diagnostic tools and therapy options, quarantine and social distancing were applied as public health tools to limit the dissemination of the infection (3). Despite the national differences in the severity of these restrictions, people's daily life drastically changed, with devastating emotional, social as well as economic consequences (4). As such, the burden of this pandemic goes beyond the physical illness, with considerable psychosocial stressors such as reduced interpersonal contact, fear of illness, future uncertainty, and financial strain (5).

Accumulating evidence is present from previous widespread outbreaks of infectious diseases, such as the 2014–2016 Ebola virus disease outbreak (6) or the 2009–2010 H1N1 influenza outbreak (7), that infectious diseases outbreaks are associated with psychological distress and mental health symptoms that have implications that continue far beyond the duration of the outbreak (8). Specifically for the SARS outbreak in 2002–2004, a retrospective cohort study in SARS survivors revealed a cumulative incidence of DSM-IV psychiatric disorders of 58.9%, and a point prevalence for any psychiatric disorder of 33.3% at 30 months post-SARS infection (9).

Health-related quality of life (HRQoL) is a patient-centered outcome to evaluate a person's overall physical, emotional and social wellbeing in one outcome measure (10, 11). In populations with persisting problems, among which survivors of infectious diseases, a decreased HRQoL is often reported (12–14). Persons with persisting pain for several months [i.e., the presence of chronic pain (15)], such as patients with chronic low back and leg pain or patients with fibromyalgia, also suffer from a poor HRQoL (16, 17). Due to the presence of pain, fatigue and dyspnea in COVID-19 infected persons, indicating symptoms that persist after the resolution of acute COVID-19 infection (18), it is our hypothesis that HRQoL of COVID-19 survivors will be in line with values reported by chronic pain patients. A systematic review was recently performed to investigate HRQoL in post-COVID-19 infected persons after discharge or recovery, with results suggesting female sex, an older age, the presence of co-morbidities, intensive care unit admission, prolonged intensive care unit stay and mechanical ventilation as factors that were most frequently associated with decreased levels of HRQoL (19). The time of assessment broadly varied from 12.76 days up to 6 months after discharge from the hospital or recovery, with a limited number of long-term large scale studies (19).

Therefore, the first aim of this study was to perform an in-depth evaluation of the HRQoL of post-COVID-19 infected persons in Belgium. The second aim was to compare the HRQoL of these persons with a normative population and with patients with chronic pain to evaluate the full spectrum of HRQoL.

## Materials and methods

### Study participants and reference data

Data from persons post-COVID-19 infection state were collected through a cross-sectional online survey with a convenience sample. The survey population comprised all Dutch speaking adults, residing in Belgium, while the sampling frame consisted of all post-COVID-19 infected persons who were active on LinkedIn, Facebook and Instagram between June 4th and August 22th, 2021. Additionally, personal contacts of the authors who were infected with COVID-19 were asked to complete the online survey. The complete details about this survey are presented in previous work (20). The study protocol of this cross-sectional study was approved by the central ethics committee of Universitair Ziekenhuis Brussel (B.U.N. 1432021000484). The study was registered on https://clinicaltrials.gov (NCT04912778).

Reference data for HRQoL were taken from the normal population records for Belgium (21) and from the Discover registry for chronic pain patients, which was also conducted in Belgium (22). Normal population records were obtained through the European Study of the Epidemiology of Mental Disorders survey, which was performed in six European countries among which Belgium in non-institutionalized adult persons (21). In the Discover registry, patients with Persistent Spinal Pain Syndrome Type 2 (PSPS-T2), were included. According to the ICD-11 criteria (15), these patients could be categorized as suffering from chronic secondary pain syndrome, and more specifically from chronic post-surgical pain (2nd level diagnosis) with chronic pain after spinal surgery (3th level diagnosis) (23). Patients all had a pain intensity of ≥5/10 for leg and/or back pain for a period of at least 6 months. The Discover registry was approved by the central ethics committee of Universitair Ziekenhuis Brussel (B.U.N. 143201629180), and registered on clinicaltrials.gov (NCT02787265).

The selected reference data was stratified by age category and sex, which allows matching with the current study population of persons after COVID-19 infection.

### Outcome measurements

The online survey consisted of three validated questionnaires [presented to participants in a random (arbitrary) order] to evaluate the functional status (post-COVID-19 Functional Scale), HRQoL (EuroQol with five dimensions) and symptoms of central sensitization (Central Sensitization Inventory). Survey data were collected through LimeSurvey (web application, https://www.limesurvey.org/). The survey took around 10 min to complete. In this study, we only focused on HRQoL, measured with the EuroQol with five dimensions and three levels (EQ5D-3L) (24), as a generic measure of health for clinical and economic appraisal (25). The EQ5D-3L consists of two separate parts, namely a descriptive system and a visual analog scale (VAS). The descriptive system contains five dimensions: mobility, self-care, usual activities, pain/discomfort, anxiety/depression, with three response levels per dimension. The scores on the five dimensions of the EQ5D were converted into a single index value for all health states (Belgian value set), with a range from < 0 (where zero is a health state equivalent to death; negative values are valued as worse than death) to one (perfect health) (26), with the help of an existing Shiny App calculator, specifically focused on calculating the index score from EQ5D dimension scores (https://fragla.shinyapps.io/shiny-eq5d/). In the second part of the questionnaire, a standard vertical 20-cm VAS is implemented to record an individual's rating for their current HRQoL state. The EuroQol with five dimensions is valid, reliable and responsive in the assessment of patients with several chronic pain conditions (27–29).

### Statistical analysis

All analyses were performed in R Studio version 1.4.1106 (R version 4.0.5). *P*-values of 0.05 or less were considered statistically significant. Descriptive statistics were provided as means with corresponding standard deviation (SD) or absolute number of observations with corresponding percentage. Two-sample *t*-tests were performed to evaluate whether the mean index score and mean VAS score were different for males compared to females. One-way ANOVA testing was applied to evaluate the effect of age on index scores and VAS scores, with corresponding Tukey HSD test for *post-hoc* comparisons. Age was categorized in all analyses in the following seven categories: 18–24, 25–34, 35–44, 45–54, 55–64, 65–74, and ≥75 years.

The HRQoL scores from persons post-COVID-19 infection were compared to scores from a normative population and patients with PSPS-T2 to evaluate the full spectrum of HRQoL ranging from a normative population on the one side toward a well-known chronic pain population with poor HRQoL on the other side. Two-way analyses of variance were calculated to explore the effect of condition (COVID-19, normative or PSPS-T2) and sex on index scores and VAS scores. Independence was assumed by design, without formal testing.

Additionally, the five dimensions of the EQ5D were also evaluated separately to gain more insight into the potential differences between the three populations. Observed counts and expected counts under independence were calculated per response level and condition. To test for independence, the Pearson and likelihood-ratio chi-squared tests were used to compare the expected frequencies with the observed frequencies. Subsequently, standardized Pearson residuals were calculated to identify specific cells that have a lack of fit (i.e., a cell-by-cell comparison between the observed and expected counts) and presented in mosaic plots (30). A standardized Pearson residuals that exceeds about two or three in absolute value indicates a lack of fit of the null hypothesis of independence in that cell (31). Loglinear models were calculated for each dimension, with count data for the three response levels, per population. A combined forward and backward model fitting procedure was employed to create homogeneous models. Based on the expected counts from the fitted models, odds ratios were calculated with corresponding Wald 95% confidence intervals. For each dimension, the ratio of no problems compared to problems (combining the remaining response levels) for post-COVID-19 persons compared to normative controls and for post-COVID-19 persons compared to PSPS-T2 patients was calculated. Additionally, the odds ratio for extreme problems compared to the other response levels was calculated for post-COVID-19 persons vs. normative controls and for post-COVID-19 persons vs. PSPS-T2 patients.

## Results

### Demographic statistics of persons post-COVID-19 infection

Based on the cross-sectional evaluation of the HRQoL in COVID-19 infected persons, the EQ5D-3L was completed by 547 respondents and the EQ5D VAS by 537 persons. These respondents were infected with COVID-19 between January 22th, 2020 and July 25th, 2021. The mean time between the infection and the time of completing this survey was 287 days (SD: 150). Seventy-five males (13.7%) and 472 (86.3%) females completed the questionnaire. Respondents had a mean age of 46.6 (SD: 11.5) years.

### HRQoL in post-COVID-19 infected persons

**Figure 1:**
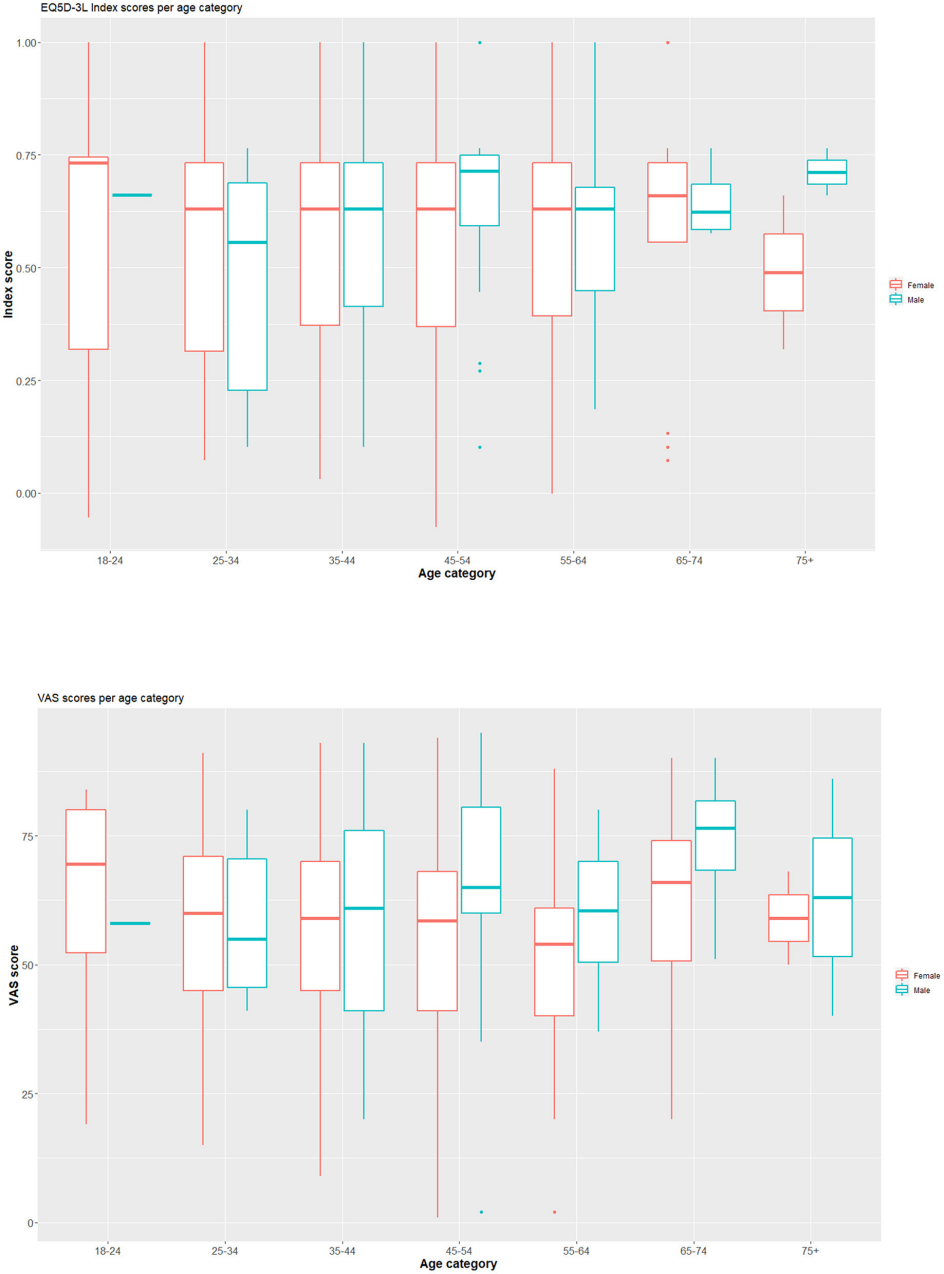
Boxplots presenting the index score and VAS score for COVID-19 infected persons, separated by sex and age category. Index scores are presented in the **upper row**, VAS scores in the **lower row**. On the plot, median values are represented by the horizontal line within the box; the latter indicating the first and third quartiles.

For the mobility component of the EQ5D-3L, 57.04% of the respondents had no problems, while 40.22% had some problems. For the self-care component, 86.84% reported no problems. For usual activities, the majority of persons reported some problems (62.71%). Moderate pain or discomfort was indicated by 74.59% and extreme pain or discomfort by 14.99%. For anxiety/depression, 57.40% indicated not being anxious or depressed. The mean EQ5D-3L index score was 0.57 (SD: 0.23) and EQ5D VAS mean score was 56.6 (SD: 18.2) (Figure 1). Table 1 presents component scores, index scores and VAS scores for all respondents.

**Table 1:** The absolute number of respondents per level for the five EQ5D dimensions, the mean index scores, and mean VAS scores for persons after COVID-19 infection.

**Dimension**	**Levels**	**All respondents** **(*N* = 547)**	**Males** **(*N* = 75)**	**Females** **(*N* = 472)**
Mobility	No problems	312 (57.04%)	47 (62.67%)	265 (56.14%)
	Some problems	220 (40.22%)	28 (37.33%)	192 (40.68%)
	Confined to bed	15 (2.74%)	0 (0.0%)	15 (3.18%)
Self-care	No problems	475 (86.84%)	66 (88%)	409 (86.65%)
	Some problems	68 (12.43%)	9 (12%)	59 (12.5%)
	Unable	4 (0.73%)	0 (0.0%)	4 (0.85%)
Usual activities	No problems	96 (17.55%)	20 (26.67%)	76 (16.10%)
	Some problems	343 (62.71%)	40 (53.33%)	303 (64.19%)
	Unable	108 (19.74%)	15 (20%)	93 (19.70%)
Pain or discomfort	No	57 (10.42%)	10 (13.33%)	47 (9.96%)
	Moderate	408 (74.59%)	55 (73.33%)	353 (74.79%)
	Extreme	82 (14.99%)	10 (13.33%)	72 (15.25%)
Anxiety/depression	No	314 (57.40%)	41 (54.67%)	273 (57.84%)
	Moderate	210 (38.39%)	33 (44%)	177 (37.5%)
	Extreme	23 (4.20%)	1 (1.33%)	22 (4.66%)
Index score		0.57 (SD: 0.23)	0.60 (SD: 0.23)*	0.56 (SD: 0.23)*
VAS scores		56.6 (SD: 18.2)	61.8 (SD: 18.5)^∧^	55.8 (SD: 18.1)^∧^

^*^Two sample t-test indicated that the mean index score in males was not significantly different from the mean index score in females (t = −1.14, p = 0.26). ^∧^Two sample t-test indicated that the mean VAS score in males was significantly different from the mean VAS score in females (t = −2.56, p = 0.01). N, number; SD, standard deviation.

The mean index score in males (0.60) was not significantly different from the mean index score in females (0.56) (*t* = −1.14, *p* = 0.26). The mean VAS score in males (61.76) was significantly different from the mean index score in females (55.82) (*t* = −2.56 *p* = 0.01, 95% CI for the mean −1.34 to −10.53). Based on one-way ANOVA testings, there was no significant effect of age category on the index score at the 5% level [*F*_(6, 540)_ = 0.384, *p* = 0.89], nor on the VAS score [*F*_(6, 530)_ = 1.425, *p* = 0.20].

### HRQoL in post-COVID-19 persons, normative controls, and patients with PSPS-T2

**Figure 2:**
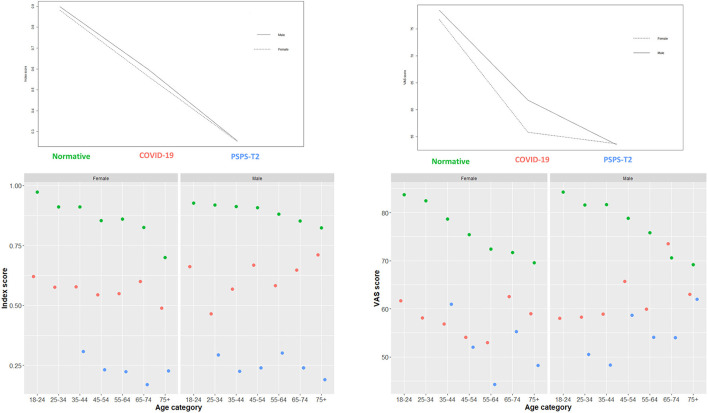
Interactions plots with condition, sex and EQ5D index scores and EQ5D VAS scores. These plots provide a general insight in the distribution of the data. Conditions consists of normative controls (green dots), persons after COVID-19 infection (red dots) and patients with Persistent Spinal Pain Syndrome Type 2 (blue dots).

A two-way analysis of variance was conducted on the influence of condition and sex on the EQ5D index scores and VAS scores (Figure 2). The main effect of sex was not statistically significant (*F* = 2.75, *p* = 0.09). The main effect of condition was statistically significant (*F* = 2,523.49, *p* < 0.01). Tuckey *post-hoc* testing indicated that the mean index score for the normative controls was significantly higher than the mean index score for COVID-19 infected persons [mean difference of 0.31 (95% from 0.29 to 0.33), *p* < 0.01]. The mean score of PSPS-T2 patients was significantly lower than the score of COVID-19 infected persons [mean difference of −0.31 (95% from −0.29 to −0.33), *p* < 0.01] and the normative controls [mean difference of −0.63 (95% CI from −0.61 to −0.65), *p* < 0.01]. For the index score, no statistically significant interaction effect was found between condition and sex [*F*_(2, 1, 561)_ = 0.791, *p* = 0.45].

**Figure 3:**
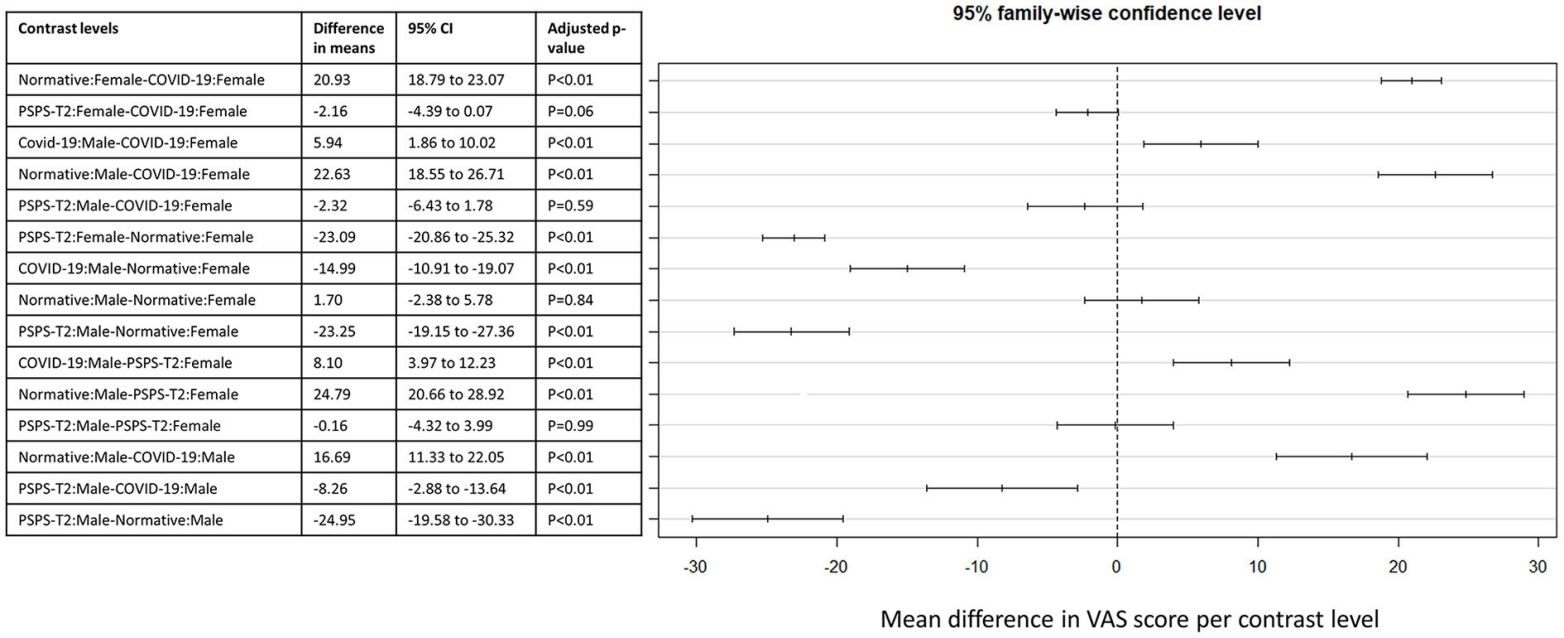
*Post-hoc* comparison between EQ5D VAS scores for the interaction effect between condition and sex. The different levels of this interaction are presented in the table with the corresponding difference in means and 95% confidence interval. A visual presentation of these effects is presented on the **right side** of the figure.

For EQ5D VAS scores, a statistically significant interaction effect was found between condition and sex [*F*_(2, 1, 535)_ = 4.719, *p* = 0.009]. The main effect of sex (*F* = 9.22, *p* = 0.002) and the main effect of condition were also statistically significant (*F* = 643.57, *p* < 0.01). The significant effects are presented in Figure 3. Tuckey *post-hoc* testing indicated that the mean VAS score for female normative controls was higher than for female COVID-19 persons [mean difference of 20.93 (95% from 18.79 to 23.07), *p* < 0.01]. Additionally, the mean VAS score was higher for male normative controls compared to male COVID-19 persons [mean difference of 16.69 (95% CI from 11.33 to 22.05), *p* < 0.01]. The mean score for female PSPS-T2 patients was lower than for female normative controls [mean difference of −23.09 (95% CI from −20.86 to −25.32), *p* < 0.01]. Finally, the mean VAS score for male PSPS-T2 patients was lower than for male COVID-19 persons [mean difference of −8.26 (95% CI from −2.88 to −13.64), *p* < 0.01] and lower than for normative controls [mean difference of −24.95 (95% CI from −19.58 to −30.33), *p* < 0.01].

### Scores on the five dimensions of the EQ5D in persons post-COVID-19 infection, normative controls, and patients with PSPS-T2

**Figure 4:**
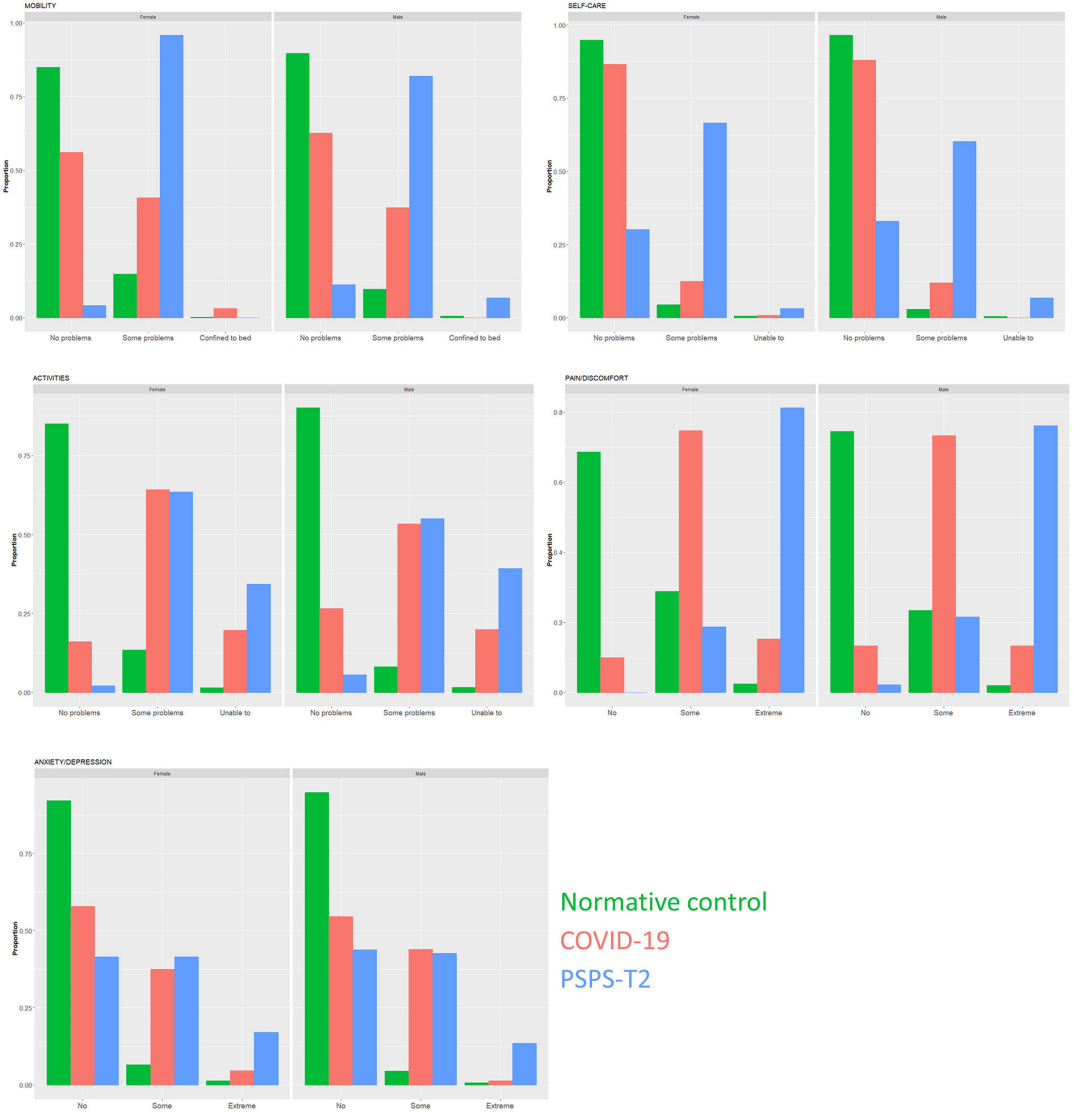
Bar plots of the proportion of response levels for each of the 5 dimensions of the EQ5D, separated by condition and sex. For each dimension, respondents could select three response levels (no problems, some problems, or extreme problems). Conditions consists of normative controls (green bars), persons after COVID-19 infection (red bars) and patients with Persistent Spinal Pain Syndrome Type 2 (blue bars). The **left frame** presents the responses for females, the **right frame** for males.

For each dimension of the EQ5D, the percentage of persons with no problems, some problems and extreme problems was calculated per population and per sex. The proportions of response levels are presented in Figure 4, for each of the 5 dimensions separately. For mobility, the null hypothesis of independence is rejected (χ^2^ = 785.48, *df* = 4, *p* < 0.01; *G*^2^ = 685.34, *p* < 0.01). Based on the standardized residuals (Table 2), there were less persons post-COVID-19 with no problems and more post-COVID-19 persons with moderate problems and confined to bed, as expected under independence.

**Table 2:** A cell-by-cell comparison of the observed frequencies and expected frequencies under independence per response level of the dimensions of the EQ5D in persons post-COVID-19 infection, normative controls, and patients with PSPS-T2.

		**Condition**		
**Dimension**	**Response level**	**COVID-19**	**Normative control**	**PSPS-T2**
Mobility	No problems	312	2,105	14
		*423*	*1,865*	*143*
	Some problems	220	296	165
		*118*	*522*	*40*
	Confined to bed	15	10	6
		*5*	*24*	*2*
Self-care	No problems	475	2,308	58
		*495*	*2,180*	*166*
	Some problems	68	90	117
		*48*	*211*	*16*
	Unable to	4	13	9
		*4*	*20*	*1*
Activities	No problems	96	2,112	7
		*385*	*1,699*	*130*
	Some problems	343	261	110
		*124*	*548*	*42*
	Unable to	108	38	68
		*37*	*164*	*13*
Pain/discomfort	No	57	1,725	2
		*311*	*1,369*	*104*
	Some	408	632	37
		*187*	*826*	*63*
	Extreme	82	54	145
		*49*	*216*	*16*
Anxiety/depression	No	314	2,253	78
		*461*	*2,030*	*154*
	Some	210	133	77
		*73*	*322*	*24*
	Extreme	23	25	28
		*13*	*58*	*4*

Observed frequencies are underlined, while expected frequencies under independence are presented in italic. If the variables were truly independent, there would only be a small discrepancy between the observed and expected counts. If there is a large discrepancy, than there is a lack of fit of the null hypothesis of independence in that specific cell.

For self-care, we could reject the null hypothesis of independence (χ^2^ = 828.07, *df* = 4, *p* < 0.01; *G*^2^ = 480.90, *p* < 0.01). There were slightly more post-COVID-19 infected persons with some problems than expected (Table 2). There were less PSPS-T2 patients with no problems for selfcare and less normative persons with some self-care problems.

Regarding activities, statistically significant independence tests were found (χ^2^ = 1,554.6, *df* = 4, *p* < 0.01; *G*^2^ = 1,480.28, *p* < 0.01). There were more post-COVID-19 persons and PSPS-T2 patients with some problems and inabilities to perform activities compared to what was expected under independence (Table 2). For normative controls, there were less persons with some problems and less persons with inabilities to perform activities.

For the dimension of pain and discomfort, independence between condition and response levels was rejected (χ^2^ = 1,863.6, *df* = 4, *p* < 0.01; *G*^2^ = 1,410.67, *p* < 0.01). The number of post-COVID-19 infected persons with some and extreme pain is higher than expected, as is the number of PSPS-T2 patients with extreme pain (Table 2). There were less normative controls with some or extreme pain than expected.

For the dimension of anxiety/depression, the null hypothesis of independence was rejected (χ^2^ = 740.53, *df* = 4, *p* < 0.01; *G*^2^ = 592.51, *p* < 0.01). Based on the standardized residuals, there were less post-COVID-19 persons and PSPS-T2 patients with no symptoms of anxiety/depression than expected (Table 2). There were more COVID-19 persons and PSPS-T2 patients with some anxiety/depression than expected. For the normative persons, more persons reported no anxiety than expected.

For each dimension, odds ratios were calculated based on the expected counts from the loglinear models and presented in Table 3. For each dimension, the odds of having no problems were lower if you are a post-COVID-19 infect person compared to a normative control. Additionally, the odds of having no problems were significantly higher for post-COVID-19 infected persons compared to PSPS-T2 patients for all dimensions [OR 16.22 (95% CI from 9.17 to 28.69) for mobility, OR 14.33 (95% CI from 9.63 to 21.33) for self-care, OR 5.41 (95% CI from 2.46 to 11.89) for activities, OR 10.58 (95% CI from 2.56 to 43.80) for pain/discomfort and OR 1.81 (95% CI from 1.29 to 2.54) for anxiety/depression]. Except for self-care, the odds of having the highest response level (i.e., most problems) was significantly higher for post-COVID-19 infected persons compared to normative controls [OR 6.77 (95% CI from 2.15 to 21.27) for mobility, OR 15.36 (95% CI from 9.44 to 24.98) for activities, OR 7.70 (95% CI from 4.65 to 12.73) for pain/discomfort and OR 4.19 (95% CI from 2.08 to 8.44) for anxiety/depression]. For all dimensions, except for mobility, the odds of having the highest response level was significantly lower for post-COVID-19 persons compared to PSPS-T2 patients.

**Table 3:** Odds ratios with corresponding 95% confidence interval for the five dimensions of the EQ5D.

**Dimension**	**Comparison**	**COVID-19 vs. normative controls**	**COVID-19 vs. PSPS-T2**
Mobility	No problems vs. problems*	0.19 (0.11–0.34)	16.22 (9.17–28.69)
	Confined to bed vs. not confined to bed^∧^	6.77 (2.15–21.27)	0.84 (0.32–2.20)
Self-care	No problems vs. problems*	0.29 (0.19–0.46)	14.33 (9.63–21.33)
	Unable to vs. able to^∧^	1.36 (0.37–5.03)	0.14 (0.04–0.47)
Activities	No problems vs. problems*	0.03 (0.01–0.07)	5.41 (2.46–11.89)
	Unable to vs. able to^∧^	15.36 (9.44–24.98)	0.42 (0.29–0.61)
Pain/discomfort	No pain vs. presence of*	0.05 (0.01–0.19)	10.58 (2.56–43.80)
	Extreme vs. no or some^∧^	7.70 (4.65–12.73)	0.05 (0.03–0.07)
Anxiety/depression	No vs. presence of*	0.09 (0.06–0.14)	1.81 (1.29–2.54)
	Extreme vs. no or some^∧^	4.19 (2.08–8.44)	0.24 (0.14–0.43)

In all calculations, COVID-19 persons are considered as the reference with no problems as reference (*) or the highest degree of problems as reference (^∧^). PSPS-T2, Persistent Spinal Pain Syndrome Type 2.

## Discussion

This study performed an in-depth evaluation of the HRQoL of persons post-COVID-19 infection with a mean time of 287 days (SD: 150) after the infection, indicating the setting of post-COVID syndrome (18). The mean EQ5D-3L index score in this population was 0.57 (SD: 0.23). When evaluating the response scores on the different dimensions of the EQ5D, this rather low score seems to be mainly affected by problems with activities and pain/discomfort. In total, 89.58% of the post-COVID-19 infected persons reported some or extreme pain and 82.45% indicated some or serious limitations when performing usual activities. Respectively, 42.96 and 42.59% indicated some or severe problems with mobility and anxiety/depression. Self-care was preserved in most post-COVID-19 infected persons, whereby only 13.16% indicated problems.

The pandemic disrupted not only social contacts of the general population, but also induced fear, stress, financial concerns, and worries about health, eventually leading to a lower HRQoL (3, 32). In Belgium, HRQoL was measured in 2099 individuals during the first 8 weeks of the coronavirus lockdown and before the COVID-19 preventive measures (33). An index score of 0.82 (95% CI from 0.80 to 0.84) was revealed before COVID-19 measures and a score of 0.79 (95% CI from 0.77 to 0.81) during the COVID-19 measures, for the general population (33). Similarly for the VAS, mean scores of 72.9 (95% CI from 71.0 to 74.0) were revealed during the pandemic and 74.5 (95% CI from 72.6 to 76.3) before the COVID-19 measures (33). The mean index score (0.57) and VAS score (56.6) in our sample of persons post-COVID-19 infection seemed considerably lower than those of normative persons, even during the lockdown. This observation was confirmed by the two-way analyses of variance which clearly denoted a significantly lower index and VAS score for post-COVID-19 infected persons compared to normative controls. This result is in contrast to the Norwegian situation where EQ5D index scores were not different from those of the general population (34). More specifically, the sample of persons post-COVID-19 infection had an index score of 0.82 (SD: 0.17) (34), considerable higher than the mean score of 0.57 (SD: 0.23) in our sample. In Norway, the proportion of persons with slight problems was higher for COVID-19 infected persons compared to normative controls for mobility and usual activities, but not for the other dimensions (34). Therefore, it seems that within Belgium, there was a higher impact of COVID-19 infection on HRQoL, whereby also pain/discomfort and psychological factors (anxiety/depression) were affected due to the infection, in addition to mobility and usual activities, the latter being dimensions which seem to have affected people in both countries. These results suggest that COVID-19 had a different impact on the HRQoL in European countries and that caution is needed when comparisons between countries are of particular interest (3).

Recently, it was recommended that policymakers and healthcare providers must urgently investigate robust strategies for improving the HRQoL in persons post-COVID-19 infection (19). The current study results strongly support this statement, since both index scores and VAS scores were significantly lower in post-COVID-19 infected persons, compared to normative persons. In some cases, acute viral illnesses hold the potential to cause both widespread and regional chronic pain (5, 35, 36), potentially leading to an increase in the number of patients with chronic pain (5). Persisting symptoms after COVID-19 infection that are often denoted are fatigue and dyspnoea (37), with fatigue being also one of the core symptoms in central sensitization (38), as underlying neurophysiological mechanism of chronic non-specific pain (39). Therefore, it could be suggested that persons post-COVID-19 infection demonstrate a clinical profile similar to that of chronic pain patients (i.e., presence of nociplastic pain) (40, 41), with central sensitization as shared underlying neurophysiological mechanism. In this study, HRQoL of post-COVID-19 infected persons was compared with HRQoL of chronic pain patients. It is commonly accepted that the multidimensional negative impact of chronic pain leads to poorer HRQoL among patients with chronic pain compared to the general population (10, 42) and even in comparison to patients with other long-term conditions (43). Compared to patients with PSPS-T2, HRQoL of post-COVID-19 infected persons was significantly higher. Therefore, it may be suggested that persons after COVID-19 infection seem to position themselves in between the normative HRQoL of a healthy population and the poor HRQoL of chronic pain patients. The fact that HRQoL is not as negatively influenced as in chronic pain patients, does not mean that treatment should not focus on improving HRQoL. It remains to be seen, but it may be possible that persons post-COVID-19 infection will develop a similar profile as patients with PSPS-T2 if symptoms keep persisting for several years, clearly pointing out the importance of improving HRQoL in this population. Several online/mobile initiatives are postulated within literature to increase HRQoL, among which web-based psychotherapeutic interventions (44), smartphone-based pain management applications incorporating the psychological and physical management of pain (45), or eHealth combined acceptance and commitment and compassion-based self-management interventions (46). Additionally, pain education as treatment on its own or in combination with (a) physiotherapy, (b) cognitive behavioral therapy and physiotherapy, and (c) counseling and physiotherapy have been denoted as more effective than physiotherapy alone to improve health related HRQoL (47), clearly pointing toward the value of pain neuroscience education to have a positive impact on HRQoL of individual persons.

The major strength of this study is that an in-depth evaluation of HRQoL of post-COVID-19 infected persons was performed, in relation to age- and sex-adjusted reference values from the general population and values obtained from a chronic pain population, namely patient suffering from PSPS-T2 to gain a better insight in the underlying relations between HRQoL in these populations. Ideally, data from other chronic pain populations and normative persons during the COVID-19 pandemic should be added to the models to further explore the continuum of HRQoL. Therefore, the main limitation of this study is the restricted availability of freely accessible individual datasets and the differences in measures to collect HRQoL [for example EQ5D with 3 levels vs. 5 levels (48)], which have limited us to further explore the continuum of HRQoL.

## Conclusions

Health-related quality of life of persons post-COVID-19 infection is severely impacted compared to age- and sex adjusted normative controls, with mainly problems in the area of usual activities and pain/discomfort. In relation to patients with chronic pain after spinal surgery, the HRQoL of post-COVID-19 infected persons appears to be better.

## Data availability statement

The datasets presented in this article are not readily available because datasets will be available by a motived and reasonable request addressed to the corresponding author. Requests to access the datasets should be directed to lisa.goudman@vub.be.

## Ethics statement

The studies involving human participants were reviewed and approved by UZ Brussel/VUB. Written informed consent for participation was not required for this study in accordance with the national legislation and the institutional requirements.

## Author contributions

MM, AD, and LG: conceptualization and methodology. MM, RD, and LG: data analysis. All authors: interpretation of data, critical input on manuscript, and provide approval for publication of the content.

## Conflict of interest

LG is a postdoctoral research fellow funded by the Research Foundation Flanders (FWO), Belgium (project number 12ZF622N). PR reports grants and consultant fees from Medtronic, Abbott, and Boston Scientific, outside the submitted work. MM has received speaker fees from Medtronic, Nevro, and Saluda Medical. STIMULUS received independent research grants from Medtronic. The remaining authors declare that the research was conducted in the absence of any commercial or financial relationships that could be construed as a potential conflict of interest.

## Publisher's note

All claims expressed in this article are solely those of the authors and do not necessarily represent those of their affiliated organizations, or those of the publisher, the editors and the reviewers. Any product that may be evaluated in this article, or claim that may be made by its manufacturer, is not guaranteed or endorsed by the publisher.
